# Genomic Analysis Reveals Heterogeneity Between Lesions in Synchronous Primary Right-Sided and Left-Sided Colon Cancer

**DOI:** 10.3389/fmolb.2021.689466

**Published:** 2021-08-04

**Authors:** Hanqing Hu, Qian Zhang, Rui Huang, Zhifeng Gao, Ziming Yuan, Qingchao Tang, Feng Gao, Meng Wang, Weiyuan Zhang, Tianyi Ma, Tianyu Qiao, Yinghu Jin, Guiyu Wang

**Affiliations:** ^1^ Colorectal Cancer Surgery Department, The Second Affiliated Hospital of Harbin Medical University, Harbin, China; ^2^ Colorectal Cancer Surgery Department, Cancer Hospital of the University of Chinese Academy of Sciences (Zhejiang Cancer Hospital), Hangzhou, China; ^3^ Department of Surgery, The Second Affiliated Hospital of Xi’an Jiaotong University, Xi’an, China

**Keywords:** synchronous multiple primary cancer, genome, right-sided colon cancer, left-sided colon cancer, heterogeneity

## Abstract

**Background:** The synchronous primary right-sided and left-sided colon cancer (sRL-CC) is a peculiar subtype of colorectal cancer. However, the genomic landscape of sRL-CC remains elusive.

**Methods:** Twenty-eight paired tumor samples and their corresponding normal mucosa samples from 14 patients were collected from the Second Affiliated Hospital of Harbin Medical University from 2011 to 2018. The clinical–pathological data were obtained, and whole-exome sequencing was performed based on formalin-fixed and paraffin-embedded samples of these patients, and then, comprehensive bioinformatic analyses were conducted.

**Results:** Both the lesions of sRL-CC presented dissimilar histological grade and differentiation. Based on sequencing data, few overlapping SNV signatures, onco-driver gene mutations, and SMGs were identified. Moreover, the paired lesions harbored a different distribution of copy number variants (CNVs) and loss of heterozygosity. The clonal architecture analysis demonstrated the polyclonal origin of sRL-CC and inter-cancerous heterogeneity between two lesions.

**Conclusion:** Our work provides evidence that lesions of sRL-CC share few overlapping mutational signatures and CNVs, and may originate from different clones.

## Introduction

Synchronous multiple primary colorectal cancer (SM-CRC) refers to at least two primary lesions identified in a single patient. Synchronous primary right-sided and left-sided colon cancer (sRL-CC) is a peculiar subtype of SM-CRC. Previous studies have shown that genomic molecular aberration is a contributor to synchronous neoplasia (Ogino et al., 2006). However, the genomic landscape of sRL-CC remains to be elucidated.

Recently, many works have shown that solitary left-sided colon cancer and right-sided colon cancer have different biological behaviors. Clinical studies revealed that patients with right colon cancer (RCC) have a worse overall survival (Ishihara et al., 2018). Patients with metastatic left colon cancer (LCC) respond more effectively to cetuximab-based target therapy compared to those with metastatic RCC (Elez et al., 2015; Arnold et al., 2017; Tejpar et al., 2017). In addition to clinical trials, molecular studies have shown different genomic phenotypes in LCC and RCC. *TP53* and *APC* are mutated more frequently in LCC, whereas *PIK3CA*, *CTNNB1*, *ATM*, *PTEN*, and *BRCA1* are mutated more frequently in RCC (Yamauchi et al., 2012; Takahashi et al., 2016; Salem et al., 2017). The consensus molecular subtype (CMS) according to the transcriptome has divided colorectal cancer into four subtypes: CMS1 (immune activation and JAK-STAT activation), CMS2 (WNT activation, MYC activation, EGFR or SRC activation, and VEGF or VEGFR activation), CMS3 (DNA damage repair, glutaminolysis, and lipidogenesis), and CMS4 (mesenchymal transition and complement activation immunosuppression) (Guinney et al., 2015). CMS1 and CMS3 are frequent in RCC, whereas CMS2 and CMS4 are frequent in LCC (Dienstmann et al., 2017). Moreover, LCC and RCC also present different immune landscapes. For RCC, decreased infiltration of CD8^+^ T cells and Th1 cells were identified. For LCC, infiltration of CD56^high^ natural killer cells and activation of IFN-α signaling were identified (Zhang et al., 2018). Despite continuous anatomy, left-sided and right-sided colon cancer could be two sides of a coin in solitary colorectal cancer, which provokes us to explore the molecular phenotype of sRL-CC.

Previous studies had reported the heterogeneity and independent genetic origin of synchronous colorectal cancer (Cereda et al., 2016; Wang et al., 2018), but these studies have not compared the genetic phenotype according to the tumor location. In our present work, we analyzed 14 cases of sRL-CC to investigate single nucleotide variation, somatic mutation, and copy number alteration in sRL-CC patients based on whole-exome sequencing data.

## Materials and Methods

### Sample Collection

Nineteen sRL-CC patients were enrolled from March 2011 to October 2018 and five patients were excluded due to the small sample volume of the tumor tissue. The diagnosis was confirmed by two experts in the Department of Pathology from the Second Affiliated Hospital of Harbin Medical University. Fourteen patients denied hereditary history of CRC and were diagnosed with sporadic CRC. All the samples were formalin-fixed and paraffin-embedded. The splenic flexure, descending colon, and sigmoid colon were classified as the left-sided colon, while the caecum, hepatic flexure, and ascending colon were classified as the right-sided colon (Missiaglia et al., 2014). The clinicopathological data of the 14 sRL-CC were all available. All the samples were acquired with the approval of the ethics committee from the Second Affiliated Hospital of Harbin Medical University and written informed consent was obtained from all the participants.

### DNA Extraction and Whole-Exome Sequencing

The DNA was extracted from FFPE samples using a QIAamp DNA FFPE Tissue Kit (Qiagen, China), according to the manufacturer’s protocol, after each slide was reviewed by two pathological experts to ensure tumor purity was greater than 50%. The quality of DNA and contamination was evaluated on 1% agarose gels and the concentration of DNA was calculated by a Qubit DNA Assay Kit in a Qubit 2.0 Fluorometer (Invitrogen, China). Two micrograms of genomic DNA was used to prepare the captured libraries by an Agilent SureSelect Human All Exon V5 kit (Agilent Technologies, China), following the manufacturer’s recommendations. The reads library was sequenced on the Illumina Novaseq 6000 platform.

### Raw Data Processing for Calling Single Nucleotide Variants and Indels

The clean data were obtained after discarding the adapter and paired reads of the raw data from the Novaseq 6000 platform. Next, quality control was performed including reads number, error rate, and percentage of reads with average quality (>Q20 and >Q30). Burrows–Wheeler Aligner (BWA) software was used to map the paired-end clean reads to the reference genome (UCSC Human Genome Reference hg38) (Li and Durbin, 2009). Aligned reads were processed in terms of marked duplicates, realignment of indels, and base recalibration by the Genome Analysis Toolkit (GATK) (McKenna et al., 2010). Variants were identified in accordance to dbSNP (Sherry et al., 2001) and the 1000 Genomes database (Genomes Project et al., 2012), which was annotated by ANNOVAR (Wang et al., 2010). Next, SNVs and indels were identified by MuTech (Cibulskis et al., 2013) and Strelka (Saunders et al., 2012).

Based on these data, cluster analysis was conducted on 96 somatic mutational nucleotide types through nonnegative matrix factorization (Nik-Zainal et al., 2012), and three signatures were identified (Alexandrov et al., 2013a). These signatures were clustered based on 30 known signatures according to the COSMIC database to reveal the biological process of the signatures (Alexandrov et al., 2013b). The degree of similarity was assessed by the cosine similarity coefficient index.

### Mutation Signature Analysis

To investigate the predisposing genes, we analyzed germline mutation in the normal tissue. Compared with the Cancer Gene Census (CGC) database (Gerlinger et al., 2014) using an in-house algorithm, the predisposing genes were identified. To determine driver mutation genes in carcinogenesis, the mutation spectrum was aligned with published driver mutations *via* OncodriveCLUSTL (Arnedo-Pac et al., 2019) and OncodriveFM (Gonzalez-Perez and Lopez-Bigas, 2012) software referring to four databases, the CGC513 database (Futreal et al., 2004), 125 mutation genes reported by Bert Vogelstein (Vogelstein et al., 2013), SMG127 (Kandoth et al., 2013), and Comprehensive435 (Tamborero et al., 2013). A significantly mutated genes test was applied to define the SMGs in the tumor, and a mutation relation rest was applied to explore the relationship between SMGs (Dees et al., 2012).

### Phylogenetic Tress and Clonal Architecture Analysis

Phylogenetic trees analysis was performed based on the WES data. The branch and trunk lengths reflected the number of nonsynonymous mutations, which were also marked beside the branch and trunk (Gerlinger et al., 2014). In accordance with the Pyclone algorithm, clonal architecture analysis was performed (Roth et al., 2014).

### Copy Number Analysis

The copy number variants (CNVs) were evaluated by control-FREEC software based on the WES data (Boeva et al., 2012). The profiles of CNVs were calculated with alignment to the BAM data. Next, the CNVs were normalized to obtain the number of CNVs in different regions on the chromosome.

### Statistical Analyses

Statistical analyses were conducted by SPSS software (version 25.0). The comparison between the left-sided and right-sided lesions with SNP signature and TMB was performed by a two-tailed paired t test. The differences were considered to be significant at *p* < 0.05. The *p* values are described in the corresponding figure legend.

## Results

### Clinical Characteristics of Synchronous Primary Right-Sided and Left-Sided Colon Cancer

We collected 9,876 colorectal cancer patients, who were confirmed by pathological examination, in the Second Affiliated Hospital of Harbin Medical University from 2011 to 2018. A total of 7,369 patients received radical colectomy, and the samples were preserved by the formalin-fixed paraffin-embedded method. Among these patients, 67 were diagnosed with SM-CRC and 19 SM-CRC patients were identified as sRL-CC. The tumor location was evaluated by colonoscopy or computed tomography. During the surgery, the tumor location was confirmed by two experienced surgeons and recorded in the pathological reports. At last, 14 patients were enrolled for further analysis and 5 patients were excluded due to the small size of the tumor. The demographic characteristics of the 14 patients are presented in Table 1. As shown in Table 1, the median age of these patients was 69 years. Of the 14 patients, 4 were women and 10 were men. The CEA and CA-199 level was elevated (cutoff value, CEA: 5 ng/ml, CA-199: 30 U/ml) in most cases, suggesting a heavy tumor burden. As for the two lesions in most patients, the left lesions were located at the sigmoid colon and the right lesions were located at the right colon. However, in two patients, the left lesions were located at the left colon and right lesions were located at the right colon. Mucinous adenocarcinoma was identified in right-sided lesions but not in left-sided lesions. The right-sided lesions showed an advanced disease compared to the left-sided lesions according to the pathological stages and indicators, which suggested that left-sided and right-sided lesions may be at different stages of CRC development.

**TABLE 1:** Demographic characteristic of the patients in our study.

	Age	Gender	CEA (ng/ml)	CA199 (U/ml)	Location	Histology	Differentiation	Stage	Perineural invasion	Venous invasion	Lymphatic invasion
1	60	F	6.58	146.26	Right colon	TA/MA	Moderately	T_3_N_1c_M_0_	Yes	Yes	Yes
					Sigmoid colon	TA	Well/moderately	T_1_N_0_M_0_	No	No	No
2	36	F	18.71	32.57	Right colon	TA	Moderately	T_3_N_0_M_0_	No	No	No
					Sigmoid colon	TA	Moderately	T_2_N_0_M_0_	No	No	No
3	84	M	3.15	17.26	Right colon	TA/MA	Poorly	T_3_N_1a_M_0_	No	Yes	Yes
					Left colon	TA	Well	T_2_N_0_M_0_	No	No	No
4	68	M	6.85	3.22	Right colon	TA	Moderately	T_3_N_0_M_0_	No	Yes	No
					Sigmoid colon	TA	Moderately	T_3_N_0_M_0_	Yes	Yes	No
5	70	M	10.34	16.39	Right colon	VA	Moderately	T_3_N_0_M_0_	Yes	No	Yes
					Sigmoid colon	VA	Moderately	T_1_N_0_M_0_	No	No	No
6	74	F	3.65	4.06	Right colon	TA	Moderately	T_3_N_0_M_0_	No	No	No
					Sigmoid colon	TA	Moderately	T_2_N_1a_M_0_	No	No	Yes
7	83	M	1.81	34.54	Right colon	TA	Well	T_1_N_0_M_0_	No	No	No
					Sigmoid colon	TA	Well	T_1_N_0_M_0_	No	No	No
8	45	F	1.37	11.06	Right colon	TA/MA	Well/moderately	T_2_N_0_M_0_	No	No	No
					Left colon	TA	Well/moderately	T_1_N_0_M_0_	No	No	No
9	75	M	49.83	31.53	Right colon	TA	Moderately	T_3_N_0_M_0_	No	No	No
					Sigmoid colon	TA	Moderately	T_3_N_0_M_0_	No	No	No
10	84	M	0.95	1.92	Right colon	VA	Well/moderately	T_2_N_0_M_0_	No	No	No
					Sigmoid colon	TA	Moderately	T_1_N_0_M_0_	No	No	No
11	58	M	28.36	79.83	Right colon	TA	Moderately	T_3_N_0_M_0_	Yes	Yes	Yes
					Sigmoid colon	TA	Moderately	T_2_N_0_M_0_	No	No	No
12	75	M	1.66	78.52	Right colon	TA/MA	Moderately	T_3_N_0_M_0_	No	No	No
					Sigmoid colon	TA	Moderately	T_1_N_0_M_0_	No	No	No
13	60	M	3.7	43.47	Right colon	TA	Moderately	T_3_N_0_M_0_	Yes	Yes	Yes
					Sigmoid colon	TA	Moderately	T_3_N_0_M_0_	Yes	No	No
14	50	M	24.08	8.98	Right colon	MA	Poorly	T_3_N_0_M_0_	Yes	No	No
					Sigmoid colon	TA	Moderately	T_1_N_0_M_0_	Yes	No	Yes

F, female; M, male; TA, tubular adenocarcinoma; VA, villous adenocarcinoma; MA, mucinous adenocarcinoma.

### Single Nucleotide Variants Analysis of Synchronous Primary Right-Sided and Left-Sided Colon Cancer

**FIGURE 1:**
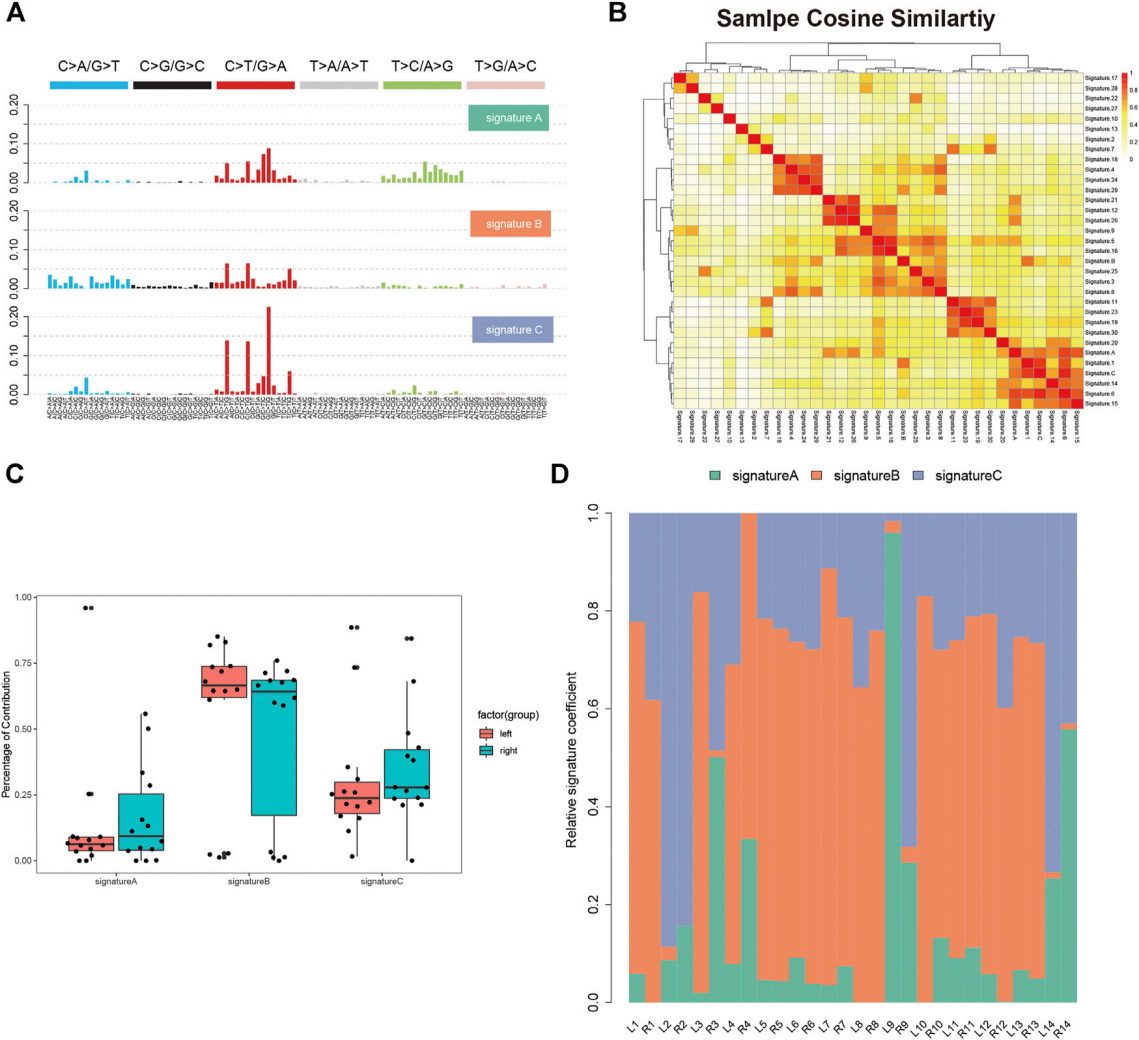
Single nucleotide variants analysis of sRL-CC. **(A)** Three SNV signatures are obtained from WES data in 14 sRL-CC patients. **(B)** Correlation analysis of three SNV signatures with known 30 signatures. The bar represents the coefficient index. **(C)** The box plot shows the percentage of the three signatures in left-sided and right-sided lesions when analyzed as a whole. Signature A: *p* = 0.722, two-tailed paired t test; signature B: *p* = 0.539, two-tailed paired t test; and signature C: *p* = 0.589, two-tailed paired t test. **(D)** The distribution of the three signatures in two lesions of each patient is presented by the heatmap.

We analyzed the single nucleotide variants (SNVs) based on the WES data of 14 paired tumor specimen and their corresponding normal mucosa. Six types of SNVs were identified in sRL-CC patients, and C>T/G>A transition was preponderant nucleotide substitution in all the samples (Supplementary Figure S1A). Then, we evaluated the percentage of each type, which were similar in left and right lesions (Supplemental Figure S1B). To explore the signatures of the SNV spectrum, we clustered SNVs to obtain three signatures (Figure 1A). In signature A, C>T/G>A and T>C/A>G transition was abundant, signature B featured C>A/G>T and C>T/G>A transition, and signature C was defined as a high level of C>T/G>A transition (Figure 1A). To investigate the function of the three signatures, we performed cosine similarity analysis according to the COSMIC database (Blokzijl et al., 2018). It revealed that signature A was mostly related to signature 6 (correlation coefficient: 0.837), which was associated with defective DNA mismatch repair. Signature B was mostly related to signature 1 (correlation coefficient: 0.725), which was associated with the biological process initiated by spontaneous deamination of 5-methylcytosine. Signature C was also mostly related to signature 6 (correlation coefficient: 0.920) (Figure 1B). To evaluate the composition of signatures in paired samples, we analyzed the left-sided and right-sided lesions as a whole. In spite of no statistical significance resulting from the small number of patients, the average percentage of signature A and C contribution was higher in right lesions, but the average percentage of signature B contribution was higher in left lesions (Figure 1C). Then, we evaluated the signatures in a single patient, respectively. For instance, signature B was dominated in L2, but signatures A and C were dominated in R2. In patient 9, signature A was dominated in the left lesion and signature C was dominated in the right lesions. There were still some special cases, such as patients 5–8 and 13, in whom the dominated signature was similar (Figure 1D). This finding suggests that distinctive mutational processes happen in the right or left lesion during carcinogenesis.

### Mutation Signature Between Lesions of Synchronous Primary Right-Sided and Left-Sided Colon Cancer

**FIGURE 2:**
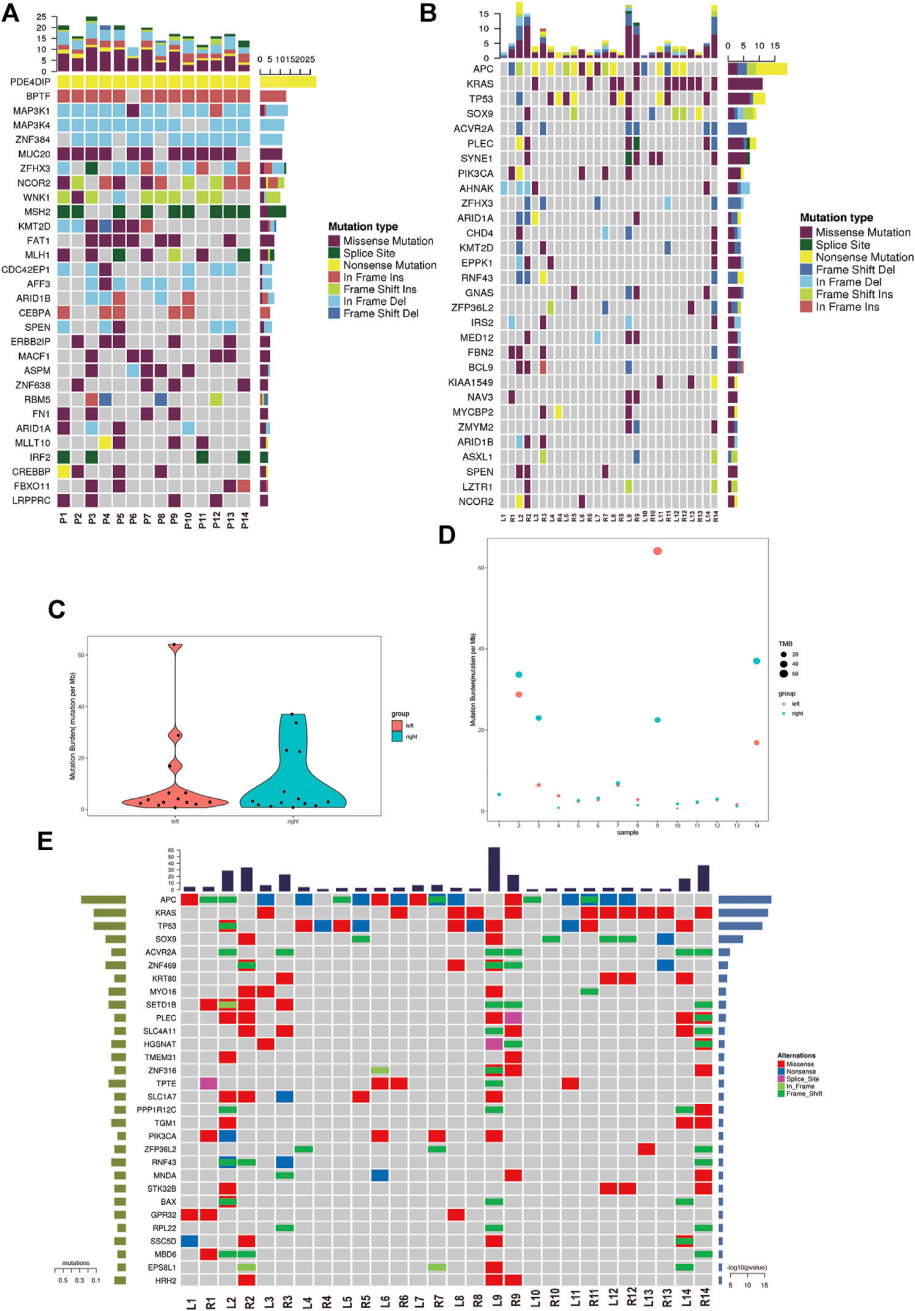
The mutational analysis of sRL-CC. **(A)** The frequent predisposing genes are presented by the heatmap. The tumor mutation burden is marked on the top of the heatmap, the name of predisposing genes is marked on the left, and the number of mutated samples is marked on the right. **(B)** The oncodriver gene mutations are presented by the heatmap. The tumor mutation burden is marked on the top of the heatmap, the name of predisposing genes is marked on the left, and the number of mutated samples is marked on the right. **(C)** The tumor mutation burden is calculated in the left-sided and right-sided lesions. **(D)** The tumor mutation burden is calculated in both lesions of each patient. **(E)** The significant mutated genes are presented by the heatmap. The tumor mutation burden is marked on the top of the heatmap.

We analyzed the germline mutation in the normal tissues of these patients to define predisposing genes using the in-house method and several frequent predisposing genes were identified. The nonsense mutation of *PDE4DIP* was found in all of 14 patients. The in-frame insertion of *BPTF* was found in 13 patients except patient 6. The in-frame deletion of *MAP3K1/4* and *ZNF384* was found in the majority of the patients. The missense mutation of *MUC20* was found in 11 out of 14 patients (Figure 2A). Cancer driver gene mutation plays a significant role in carcinogenesis, and we explored the oncodriver mutation in sRL-CC. The completely matched lesions failed to be identified in all 14 patients. Although similar in a small part, the features of cancer driver gene mutation between lesions were diversified in most patients. For example, frameshift insertion of *APC* and frameshift deletion of *TP53* was detected in L2 but not in R2, whereas missense mutation of *SOX9*, frameshift deletion of *ZFHX3*, and missense mutation of *SYNE1* were detected in R2 but not in L2 (Figure 2B). The significant mutation genes (SMGs) reflect the mutational phenotype of diseases; and then, we wanted to investigate the SMGs in all the lesions. When treated as a whole, the tumor mutation burden (TMB) was alike between left- and right-sided lesions (Figure 2C). When analyzed separately, TMB was different in patients 2, 3, 4, 8, 9, 10, and 14 and was similar in patients 5, 6, 7, 11, 12, and 13. Particularly, the TMB was almost the same in patient 1 (L: 4.1537674 vs. R: 4.1569451) (Figure 2D). Among these significant mutation genes, the *APC* mutation tended to happen in the left lesions, while *BAX*, *KRAS*, and *SOX9* mutations tended to happen in right-sided lesions. The *PI3KCA* mutation was also distributed unevenly, which was found in L2, L6, L9, R1, and R7 but not in the corresponding lesions on the other side (Figure 2E). The frequent mutation genes identified in sRL-CCs were similar to those identified in solitary colon cancer. Two studies identified eight frequent mutation genes (*APC*, *TP53*, *SMAD4*, *PIK3CA*, *KRAS*, *ARID1A*, *SOX9*, and *FAM123B*). *APC*, *TP53*, *KRAS*, and *SOX9* were the top four mutation genes in sRL-CCs (Cancer Genome Atlas, 2012; Vasaikar et al., 2019)*.* The *ACVR2A* mutation was found in hypermutation colon cancers (Vasaikar et al., 2019), which was also identified in sRL-CCs. Mutation genes in a sample may have a synergistic effect or a mutually exclusive effect. Through mutation relation rest analysis (MRT), a synergistic and mutually exclusive relationship was identified. We found that *ACVR2A* had a synergistic effect with *PLEC*, *SLC4A11*, and *CEL* (Supplementary Figure S2A). Besides, we found that the *APC* mutation was mutually exclusive with *EPS8L1*, *PPP1R12C*, *SETD1B*, *RNF43*, and *SLC4A11*. The *KRAS* mutation was mutually exclusive with *TP53*, *SLC1A7*, and *PIK3CA* (Supplementary Figure S2B). These works showed the different mutational landscape in two lesions of a single sRL-CC patient.

### Copy Number Variant Analysis of Synchronous Primary Right-Sided and Left-Sided Colon Cancer

**FIGURE 3:**
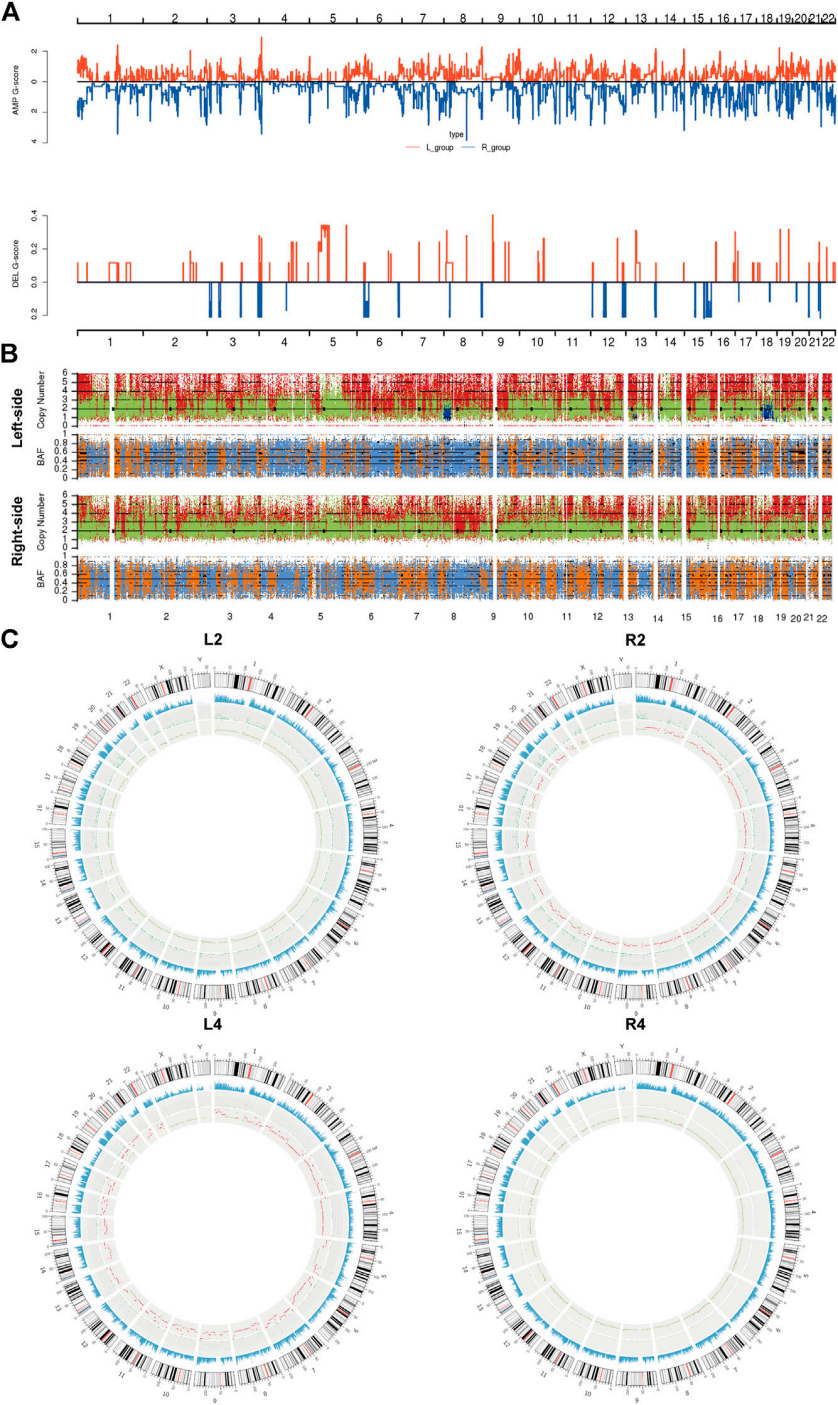
The copy number variants analysis of sRL-CC. **(A)** GISTIC analysis of left-sided and right-sided lesions. The G-score represents the degree of CNV amplification **(upper panel)** and deletion **(lower panel)**. L-group means left-sided lesions and R-group means right-sided lesions. **(B)** The upper chart illustrates the distribution of copy number variants. Red presents the CNV gain, green presents normal CNV, and blue presents CNV loss. The lower chart illustrates the distribution of β allelic frequency (BAF). Orange means the normal distribution of allele and blue means loss of heterozygosity. **(C)** Circos plots illustrate the molecular landscape of genome in patients 2 and 4 in terms of copy number variants in each patient. The first circle means the depth of sequencing, the second circle means the density of SNP insert and deletion, and the third circle means the distribution of CNVs. In the third circle, red means the CNV gains, green means normal copy number, and blue means CNV loss.

Subsequently, we assessed the CNVs in both lesions of sRL-CCs. The GISTIC curve showed that amplification of the chromosome was predominant and more deletion events were identified in left-sided lesions (Figure 3A). The distribution of somatic CNVs in left-sided lesions was also different from those in right-sided lesions (Figure 3B). Loss of heterozygosity (LOH) was evaluated by β-allelic frequency (BAF). The right-sided and left-sided lesions shared different BAF (Figure 3B). When analyzed separately, the heterogeneity stood out more clearly. As shown in the Circos plot (Figure 3C), the copy number was almost normal in L2 and R4 lesions, whereas the copy number was amplified in R2 and L4 lesions. Similar phenomena were also found in other patients of our cohort (Supplementary Figure S3). These data imply that the lesions share different patterns with respect to copy number and different stages during carcinogenesis.

### Clonal Architecture and Evolution Analysis of Synchronous Primary Right-Sided and Left-Sided Colon Cancer

**FIGURE 4:**
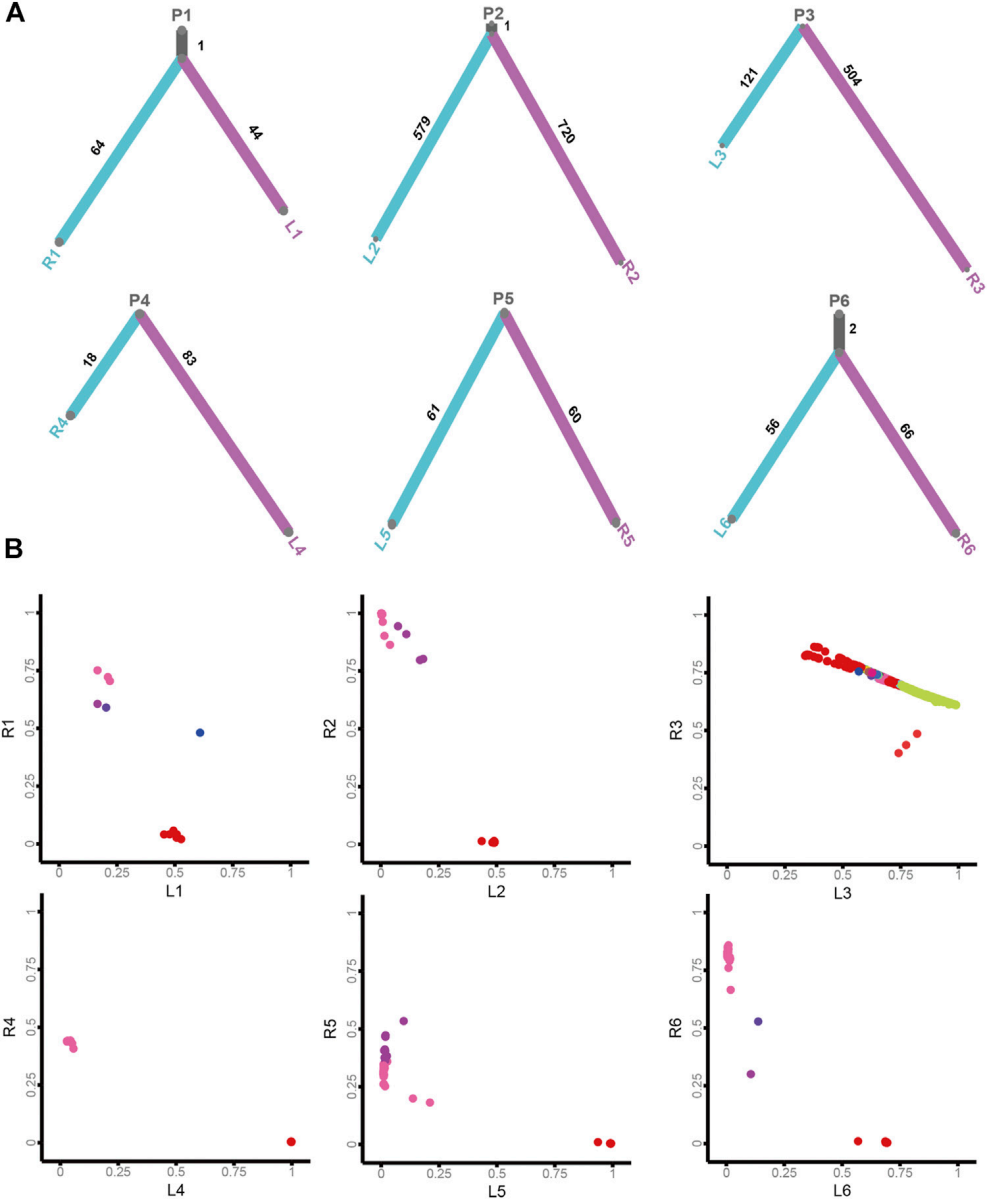
The clonal architecture and evolution analysis of sRL-CC. **(A)** The phylogenetic tress analysis of patients 1–6. The trunk represents the common mutations and the branch represents unique mutations in each lesion. The number of mutations is marked. **(B)** The scatter plot demonstrates cancer cell fraction (CCF) of the mutations in both lesions of patients 1–6. Different colors represent different clusters. The clusters located in the middle area of the plot mean shared clusters by both lesions, and those located near the X-axis and Y-axis mean unique clusters in each lesion.

To acquire insights into the origin of both lesions shaping sRL-CC tumorigenesis, phylogenetic trees analyses were constructed to evaluate the ancestral relationship of individual lesions, significantly overlapping variant sets failed to be identified in most cases in our cohort. None of the overlapping variant sets were identified in patients 3, 4, 5, 7, 8, 9, 10, 11, 13, and 14, and few overlapping variant sets were identified in patients 1, 2, and 6. But as an exception, both the lesions in patient 12 shared 53 overlapping variant sets (Figure 4A and Supplementary Figure S4A). Clonal architecture analysis revealed different clusters between two lesions. Most of the patients shared few overlapping clusters. In patients 3 and 12, relatively more sharing clusters were identified with some independent clusters. However, in patient 9, a single shared cluster was identified (Figure 4B and Supplementary Figure S4B). These data may suggest different origins and clonal architectures in the lesion of sRL-CCs.

## Discussion

In this study, we performed an unprecedented molecular characterization of both lesions and corresponding normal adjacent tissue in sRL-CCs with comprehensive data from WES. Our work revealed that heterogeneity occurred in both lesions in the same patient and each lesion was in a different stage during carcinogenesis. Our study is in line with the multi-omics analysis in single primary colorectal cancer. Vasaikar et al. reported some significant mutations such as *APC*, *TP53*, *KRAS*, *SOX9*, and *ACVR2A*, which were also found in our cohort (Vasaikar et al., 2019). The mutation of mismatch repair genes is a key molecular event in colorectal cancer (Sahin et al., 2019; Schrock et al., 2019). Our work has also identified that SNV signature is associated with defective DNA mismatch repair. All this evidence confirms that our data are accurate enough to reflect the biological process in sRL-CCs.

Recently, more and more attention has been paid to the multiple primary tumors. Grolleman et al. has reported the mutation of the base excision repair gene *NTHL1* that can trigger the development of malignancy in many organs (Grolleman et al., 2019). Our work also showed the mutational signature involved in the dysfunction of DNA mismatch repair in sRL-CCs. Our study has also highlighted predisposing genes in sRL-CC. The nonsense mutation of *PDE4DIP* was identified in all the cases in our cohort. The *PDE4DIP* gene encodes protein myomegalin and exerts its function as an anchor to sequester components to Golgi and/or centrosomes (Wang et al., 2014; Wu et al., 2016; Bouguenina et al., 2017; Yang et al., 2017). However, the role of *PDE4DIP* in cancers is poorly understood. Thus, prospective studies are needed to explore the role of nonsense mutation in *PDE4DIP* of sRL-CCs.

Above all, our study reveals heterogeneity of both lesions in sRL-CCs. We have identified different copy numbers in paired right-sided and left-sided lesions, which indicates that the lesions are in different stages of carcinogenesis. LOH is an important mechanism for the disability of tumor suppressor genes. Through the control-FREEC method (Boeva et al., 2012), the regions of LOH have been identified. The paired lesions share unmatched distribution of LOH on the chromosomes. This evidence suggests that inconsistent degrees of genome instability happen in the both lesions. Combined with the phylogenetic trees and clonal architecture analysis, we have proved the multi-origins of the paired lesions and different clonal fractions in the paired lesions. Studies based on multiple primary cancer in other organs have drawn a similar conclusion. Ma et al. reported that multicentric lesions harbor distinct oncogenic alterations and genomic heterogeneity (Ma et al., 2017). In synchronous bilateral renal cancer, the lesions in the kidneys also originate from separated clones (Linehan, 2009). In line with these previous studies, our work has shown the polyclonal origin of sRL-CC.

Limitations do exist in our study. The sample size is small and only 14 patients are included. Besides, our study is a single center, retrospective study and all the samples have to be preserved by FFPE due to the long time span. Thus, a multi-center, prospective study could collect a larger number of sRL-CC patients and fresh tumor tissue in a shorter time. Moreover, genomic analysis has trouble in reflecting the whole scenarios of sRL-CC. Systematic studies including the transcriptome, epi-transcriptome, proteome, and metabolome can provide a wider horizon for sRL-CC.

In conclusion, we performed whole-exome sequencing analyses, which are suggestive of heterogeneity between lesions and the polyclonal origin of sRL-CC. Moreover, we illustrated the genomic landscape of sRL-CC and provided an insight into the molecular pattern of sRL-CC, which could make treatment more precise and effective.

## Data Availability

The datasets presented in this article are not readily available because the Chinese laws forbid the publication the genomic data based on the Chinese population. Requests to access the datasets should be directed to the corresponding author GW, guiywang@163.com.
