# Supplementary figures and images for: Genomic Analysis Reveals Heterogeneity Between Lesions in Synchronous Primary Right-Sided and Left-Sided Colon Cancer
Source: Front Mol Biosci. 2021 Aug 4;8:689466. doi: 10.3389/fmolb.2021.689466 (PMC8371635; doi:10.3389/fmolb.2021.689466)

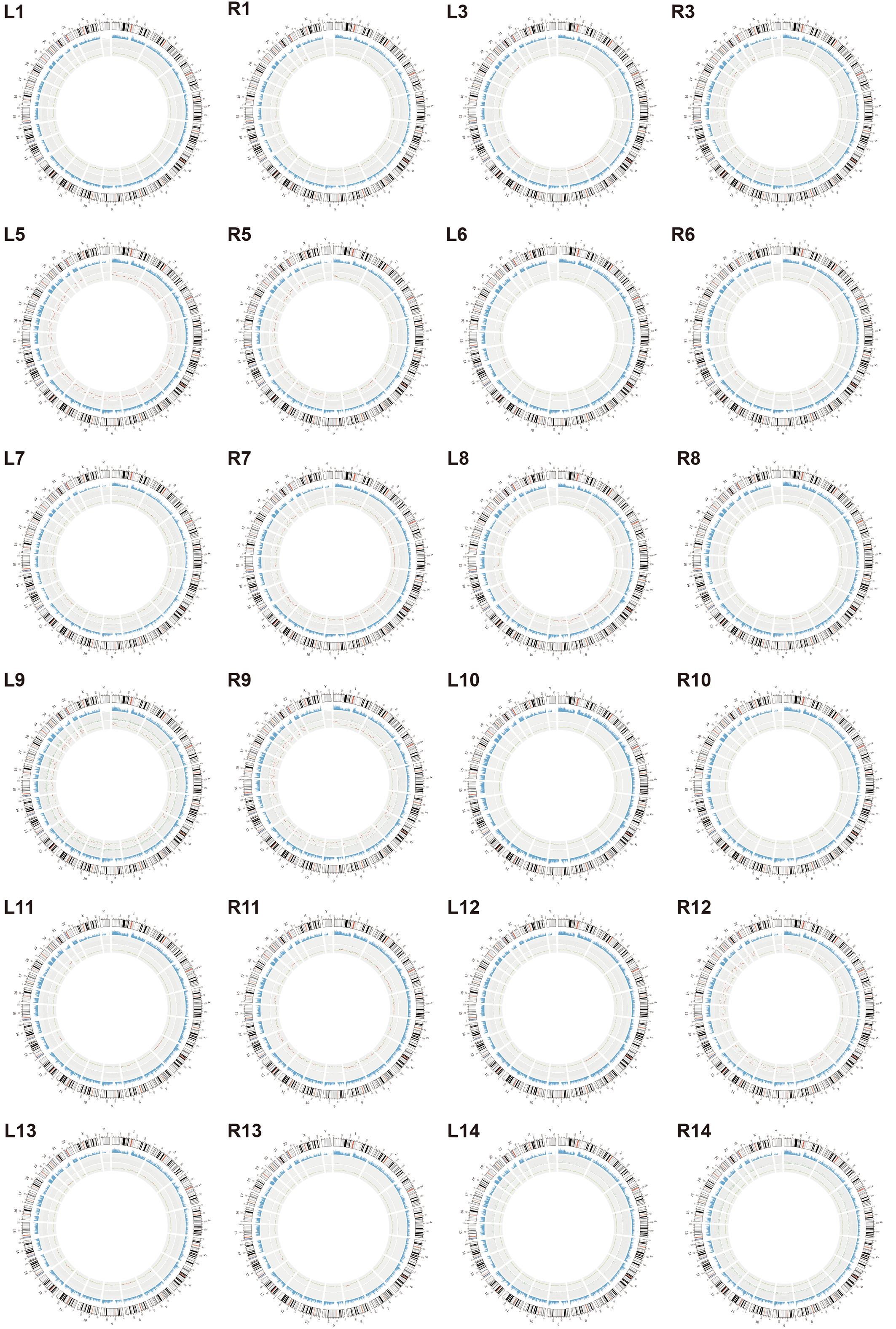

Supplement: Supplementary file 1 [file Image3.TIF]

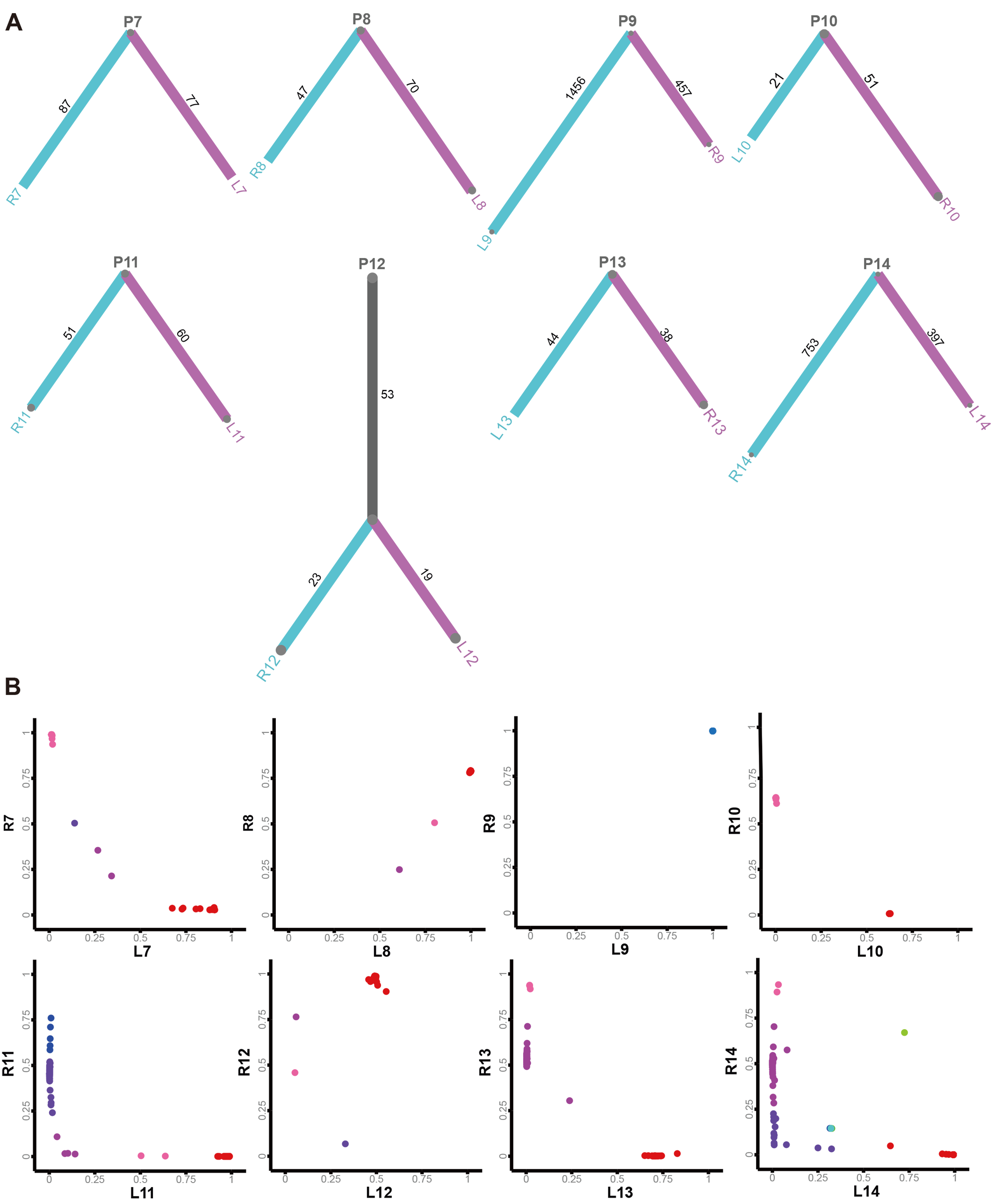

Supplement: Supplementary file 2 [file Image4.TIF]

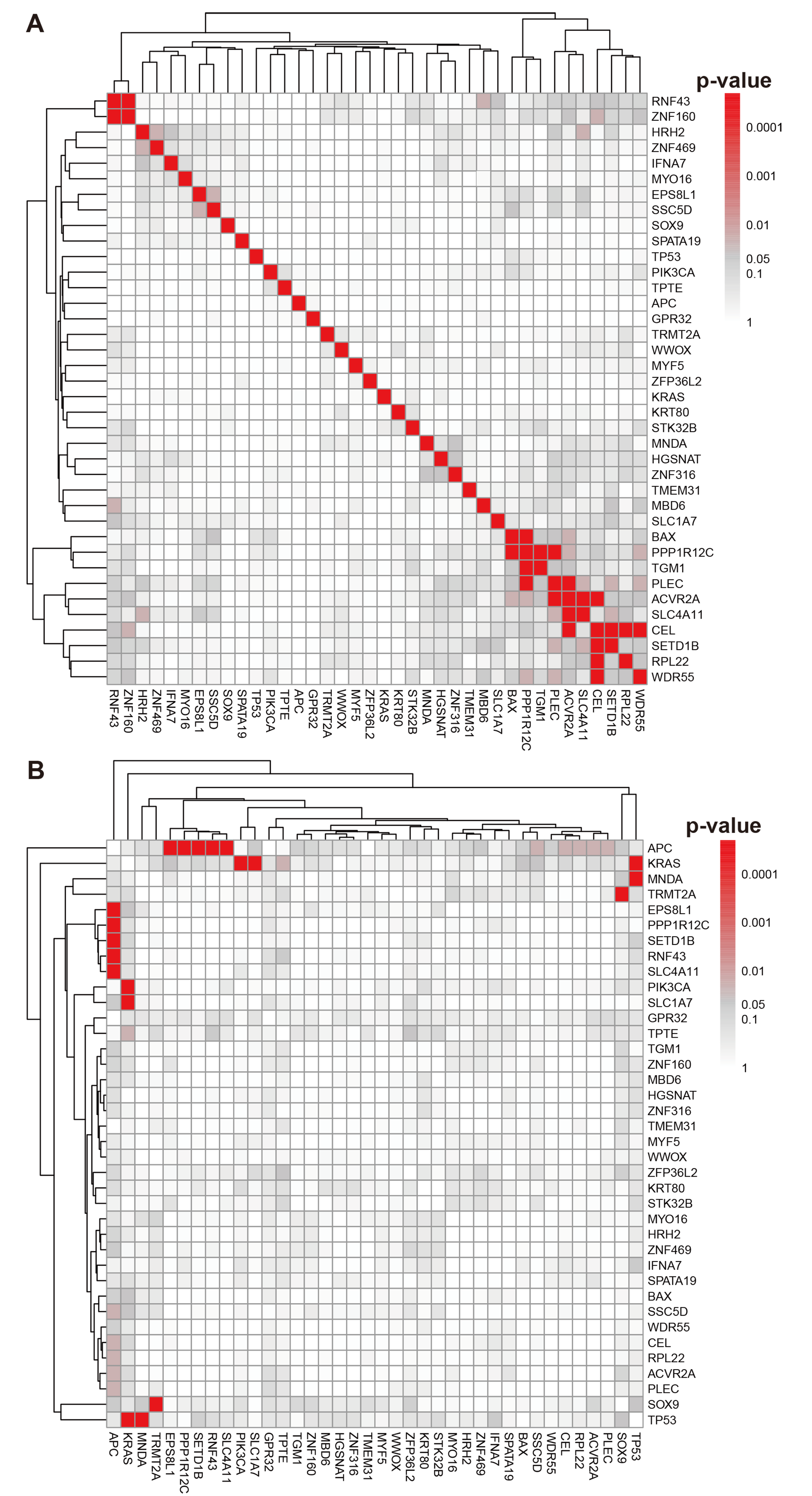

Supplement: Supplementary file 3 [file Image2.TIF]

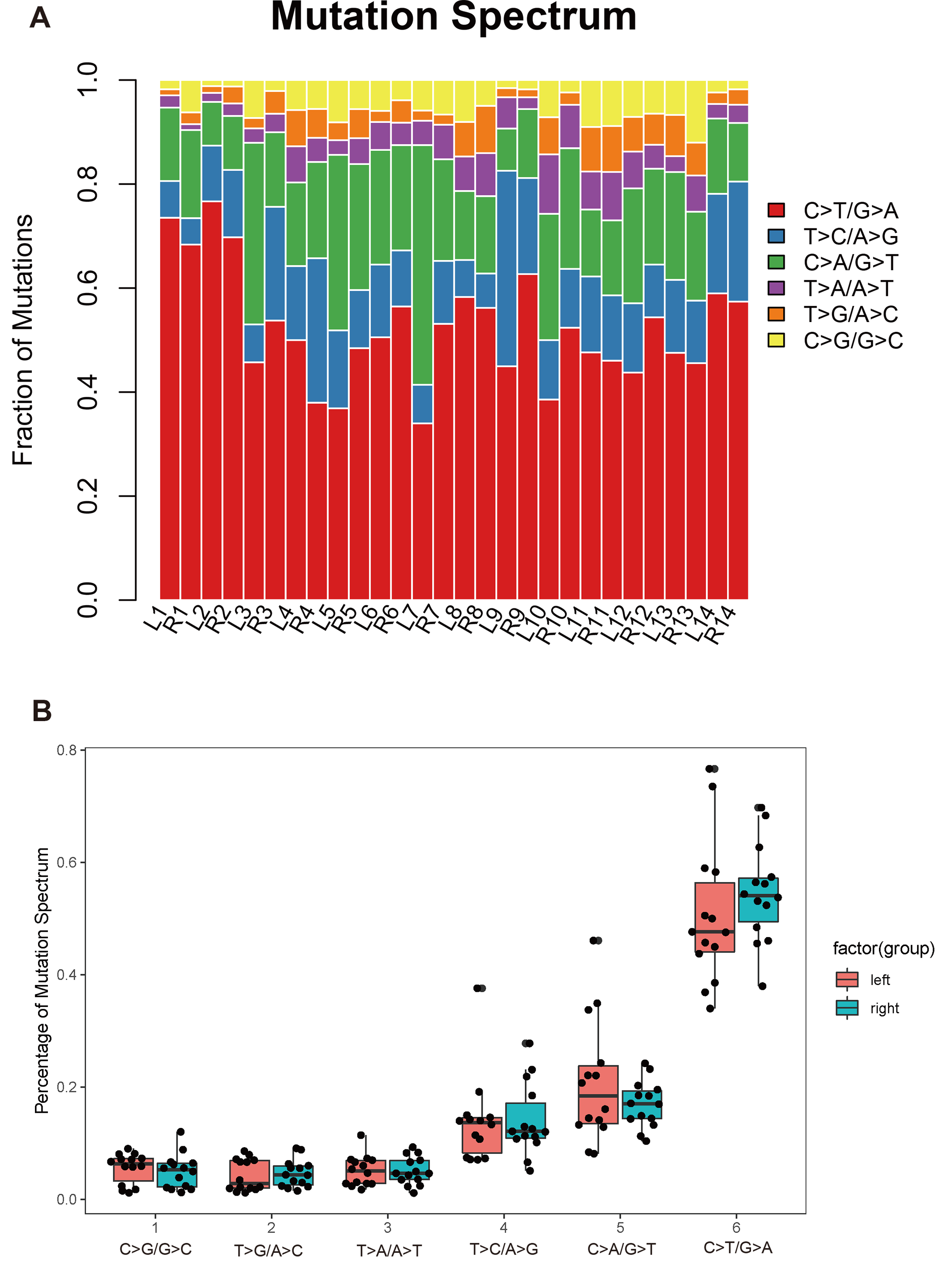

Supplement: Supplementary file 4 [file Image1.TIF]
